# Guidance on authorship with and acknowledgement of patient partners in patient-oriented research

**DOI:** 10.1186/s40900-020-00213-6

**Published:** 2020-07-02

**Authors:** Dawn P. Richards, Kathryn A. Birnie, Kathleen Eubanks, Therese Lane, Delane Linkiewich, Lesley Singer, Jennifer N. Stinson, Kimberly N. Begley

**Affiliations:** 1 Chronic Pain Network, McMaster University, Hamilton, Ontario Canada; 2 Five02 Labs Inc, Toronto, Ontario Canada; 3 Department of Anesthesiology, Perioperative and Pain Medicine, University of Calgary, Calgary, Alberta Canada; 4 Research Institute, The Hospital for Sick Children and Lawrence S. Bloomberg Faculty of Nursing, University of Toronto, Toronto, Ontario Canada

**Keywords:** Authorship, Acknowledgement, Guidance, Patient engagement, Patient involvement, Patient-oriented research, Patient partner, Publication

## Abstract

The Strategy for Patient-Oriented Research Chronic Pain Network was founded in 2016 and is a patient-oriented research network funded by the Canadian Institutes of Health Research. The Network incorporates patient partners throughout its governance and operations meaning that patient partners may contribute to research projects in ways that warrant scientific authorship as defined by the International Committee of Medical Journal Editors. The Network did a brief informal review of guidance on patient authorship in 2019, but could not find any practical documentation to guide its members on this topic. Note the term patient partner here refers to a patient (or caregiver or other person with lived experience) who is a partner or collaborator on a research team. This guidance does not address patients as participants in a research study.

This guidance has been co-written by a group of researchers and patient partners of the Chronic Pain Network in an effort to address this gap. It is intended for both researchers and patient partner audiences. This guidance is meant to facilitate conversations between researchers and patient partners about authorship and/or acknowledgement regarding research projects on which they collaborate. While the overall principles of academic authorship and acknowledgement remain unchanged, nuances for interpreting these principles through the lens of patient engagement or patient-oriented research is provided.

Teams that carry out patient-oriented research projects will require different preparation to empower all team members (researchers and patient partners) to discuss authorship and acknowledgement. To facilitate these conversations, we have included an overview of the scientific publishing process, explanation of some common terms, and sets of considerations are provided for both patient partners and researchers in determining the range of team member contribution from acknowledgement to authorship. Conversations about authorship can be difficult, even for established research teams. This guidance, and the resources discussed within it, are provided with the intention of making these conversations easier and more thoughtful.

## Plain language summary

The Chronic Pain Network is a research network that does research with patients as partners. The Network was set up in 2016 after it received peer reviewed funding from the Canadian Institutes of Health Research. The Network’s patient partners often contribute to research projects in ways that call for including them as authors on scientific papers published about the research, alongside other members of research teams. The Network could not find guidance documents about including patient partners as authors on scientific papers, so it developed this guidance to help others.

This guidance has been written by a group of researchers and patient partners to help other researchers and patient partners talk about authorship and acknowledgement for the research projects they are working on together. The same principles of authorship and acknowledgement in scientific papers apply to research teams with and without patient partners. This guidance provides more context and examples for when patient partners are part of the team. To help, an overview of the publishing process is provided and includes explanations of common terms. Two sets of considerations are provided (one for patient partners and one for researchers) to use in helping determine whether patient partners should be acknowledged or should be authors on a paper. Conversations about authorship can sometimes be hard, so this guidance document is meant to be helpful for researchers and patient partners alike.

## Background

The Canadian Institutes of Health Research (CIHR), Canada’s federal health research funding agency, created the Strategy for Patient-Oriented Research program in 2010 with the aim to support patient-oriented research in Canada. CIHR defines patient-oriented research as a continuum of research that includes patients as partners, focuses on patient-identified priorities in order to improve patient outcomes, is done by multi-disciplinary teams in partnership with relevant stakeholders, and aims to apply the knowledge generated to improve healthcare systems and practices [1]. CIHR broadly defines patients as including people with personal experience of a health issue as well as informal caregivers, including family and friends [2]. Further, CIHR stresses that patient engagement in patient-oriented research includes patients as partners via meaningful and active collaboration in governance, priority setting, and conduct of research and knowledge translation [1].

In 2016, CIHR funded a number of national chronic disease networks for a period of 5-years. One of those Strategy for Patient-Oriented Research networks was the Chronic Pain Network (herein referred to as ‘the Network’ [3]). The Network is hosted at McMaster University in Hamilton, Ontario, Canada, and represents a national chronic pain collaboration of patient partners, researchers, healthcare professionals, educators, industry and government policy advisors to direct new research, to train researchers and clinicians, and to translate research findings in to knowledge and policy. The Network aims to increase access to care for people living with pain and to speed translation of research into care. Patients are actively involved and participating as partners throughout the Network’s governance, priority setting [4], research, and knowledge translation activities. Given this high level of patient engagement across the Network, there have been instances where patient partners have been recognized as authors on scientific publications from Network-funded research projects [5] (personal communication with Dr. Dave Walton, March 5, 2020). In carrying out this work, a gap was identified in supporting Network members to determine authorship and acknowledgement contributions of patient partners.

## Main text

### Why this guidance was written

Very limited guidance specific to patient-oriented research and authorship is currently available in the published scientific or grey literature. An informal survey of the literature identified three sources of potential guidance, including: standard publisher recommendations for determining academic authorship (from the International Committee of Medical Journal Editors [ICJME]) [6, 7], articles discussing concretely how to include patients as partners in medical publications [8, 9], and a systematic review examining whether certain journals, countries or fields are more likely to include patients and members of the public as authors [10]. Although the values of patient engagement and patient partnership on research teams imply that existing ICJME recommendations for determining authorship and acknowledgement will be used, we felt that the application of these recommendations was not clear in the context of patient-oriented research and specific supplementary practical guidance was warranted. As patient-oriented research gains momentum globally, patient partner authorship on scientific publications is increasing. Given the diverse experiences of the Network, our group of researchers and patient partners felt equipped to provide some guidance to others. Discussions were undertaken within the Network and with other CIHR Strategy for Patient Oriented Research organizations. Those personal communications further illuminated the need and enthusiasm for specific authorship and acknowledgement guidance related to patient-oriented research (personal communications with Leah Getchell and Colleen McGavin in June, 2019).

This guidance has been co-developed from the idea stage to being co-written iteratively (including finalizing the responses to reviewers) by a group of the Network’s researchers and patient partners to help researchers and patient partners talk about authorship and/or acknowledgement with respect to the research projects on which they work together. Existing recommendations for academic authorship and acknowledgement are considered as relevant for patient partners as they are for other members of the research team, however patient-oriented research may require different conversations about these topics. It is important for the entire research team to be aware of patient partners’ roles and their contributions to a research project, as well as for these roles to be appropriately acknowledged and reflected in the resulting scientific literature [11, 12]. This guidance does not address patients as participants in a research study. Overall the guidance aims to help move forward meaningful and respectful patient and researcher partnerships in research. Publishing research project results together is a critical part of doing so.

### The goals of this guidance are to

1. Provide information on the steps and timeline to publish a paper in an academic journal;
2. Review existing recommendations of authorship and acknowledgement and their application within the patient-oriented research context to facilitate decision-making about patient partner authorship and acknowledgement;
3. Discuss related considerations for patient partners and researchers in patient-oriented research; and,
4. Offer practical advice based on experience of Network members whose research projects have included patient partners as authors.

Parts of this guidance are intended more for patient partners and other parts primarily for researchers. However, patient partners and researchers are encouraged to read this publication in its entirety to learn more about each other’s perspectives related to authorship and acknowledgement. It is worth noting that these topics, conversations and decisions can be controversial for research teams, even for team members that are very experienced or have worked together previously [13].

#### Steps to publishing an academic paper

**Fig. 1:**
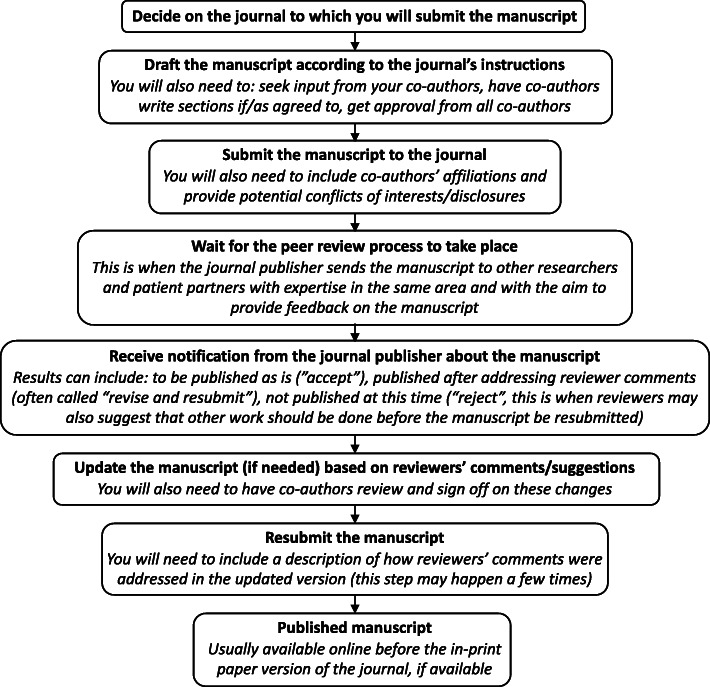
A simple overview of the academic publishing process. There are a number of steps included in writing a manuscript and having it published. Each step in the process is described to provide some detail and context about what actions are included in the step. Each step may take several weeks or months depending on how much work is involved. This process can take several months and sometimes even more than a year. Waiting for the comments from the first review of the manuscript may take months as a start

Enabling a common understanding of the publishing process for patient partners and researchers is needed for meaningful discussion of patient partner authorship. As such, it is useful to first explain the academic publishing enterprise. This is particularly relevant for patient partners who are new to scientific research and may be unfamiliar with the steps, timeline, and parts of publishing a paper (also called a manuscript). Figure 1 explains this process and reviews some of the common terms that are often used. Patient partners may be involved in any or all of the steps of publishing a paper - their role will be dependent on their role within the research team.

Common terms used in authorship and scientific publishing include:

- *Disclosure or conflict of interest:* Authors are required to disclose any relationships that might be perceived to influence the research. Conflicts of interest may include financial, commercial, legal or professional relationships, and could include consultancies, employment, advocacy groups, fees and honoraria, grants, patents, royalties and owning stocks or shares.
- *Journal:* A journal is a publication that contains scientific and medical articles written by researchers as a way to share their research, research results, and overall conclusions with others. It may be published regularly at a set time interval (e.g. weekly, monthly, etc.).
- *Impact factor:* This is a number that reflects the importance of a journal, and in general, the higher the number, the more important or influential the journal is.
- *Open access:* A term that means a journal’s contents are publicly available without a subscription fee.
- *Peer review:* In terms of academic publishing, peer review is a term used to describe when other academics and in some cases patient partners review and comment on a manuscript that has been submitted to a journal to be published. These colleagues or peers have expertise in the same area as the manuscript authors, so they are able to critically comment on the overall approach to the research, the discussion and conclusions that are drawn from the research. The process of peer review is intended to strengthen the manuscript and the work that is published.

#### Authorship and acknowledgement

The International Committee of Medical Journal Editors’ (ICMJE) document called Defining the Role of Authors and Contributors is the main resource used to create this guidance and is also widely used internationally by organizations and scientific journals to provide background information on, and criteria for, authorship and acknowledgement in scientific papers [6]. For research, authorship is a form of giving credit to people who made substantial contributions to the research results and “has important academic, social, and financial implications.” Being credited as an author means that someone has made important intellectual contributions to the work and this also holds them responsible and accountable for the work.

### Criteria on Authorship

The ICMJE recommends that authorship is based on these four criteria [6]:

1. Substantial contributions to the conception or design of the work; or the acquisition, analysis, or interpretation of data for the work; AND
2. Drafting the work or revising it critically for important intellectual content; AND
3. Final approval of the version to be published; AND
4. Agreement to be accountable for all aspects of the work in ensuring that questions related to the accuracy or integrity of any part of the work are appropriately investigated and resolved.

A useful plain language description of these authorship criteria has also been published [14]. Table 1 is included to provide further guidance for applying these four authorship criteria in the context of patient engagement and patient-oriented research.

**Table 1:** ICMJE authorship criteria explained from a patient engagement and patient-oriented research perspective. These examples are not inclusive and are meant to be demonstrative

Criterion	Application to Patient Engagement and Patient-Oriented Research
1. *Substantial contributions to the conception or design of the work; or the acquisition, analysis, or interpretation of data for the work.*	This might be the case if a patient partner is involved in the project from its start as a research idea, contributed to its design and execution plan, and contributes throughout the project. There are ways for patient partners to make substantial contributions even when they are not involved in all aspects of the research process from the outset. Patient partners may still contribute substantially to a project’s overall execution, including, but not limited to, development or selection of methods, recruitment, interpreting results, sharing results, etc. Patient partners may make substantial contributions without being trained in the scientific methodology, data analysis or interpretation. They may make these contributions through their conversations with team members about how they view the results or why they feel the results are important to patients, etc.
2. *Drafting the work or revising it critically for important intellectual content.*	Patient partners may physically contribute to writing or revising the work, or may otherwise provide intellectual content through critical and constructive comments or commentary in writing or in conversation on manuscript drafts. Drafting some of the manuscript is not necessary for making an intellectual contribution to the content.
3. *Final approval of the version to be published.*	Patient partners, as part of the authorship team, need to have reviewed and approved the manuscript for submission to be published.
4. *Agreement to be accountable for all aspects of the work in ensuring that questions related to the accuracy or integrity of any part of the work are appropriately investigated and resolved.*	Patient partners do not need to be experts in the work that was carried out (for example, statistical methods), but they do need to be accountable to the work that they did to contribute to the project as presented in the manuscript.

### Acknowledgement

Acknowledgement of patient partners and others on the research team is expected when any person contributing to the project does not meet the four criteria for authorship as explained above, but it is worthy to note their contribution. Acknowledgements may be appropriate when someone’s involvement in the project is limited to data collection, project management, and/or consultative guidance. Patient partners on a research project may or may not be involved in a project throughout its entirety. Sometimes patients’ health and life circumstances may prevent them from being a partner on the project at certain times or cause them to leave the project. In the latter case, the primary author of a paper should make a reasonable effort to contact a patient partner(s) to ensure they are comfortable with being acknowledged on a publication. Sometimes for personal reasons, patient partners may not wish to be acknowledged or included as an author. For example, this might be the case in the instance where a patient partner is from a vulnerable population and may not wish to be named on a publication. In such an instance, we would recommend that the research team formally thank their patient partner’s contribution in the acknowledgements section of the publication without using their name.

#### Considerations for patient partners and researchers

**Fig. 2:**
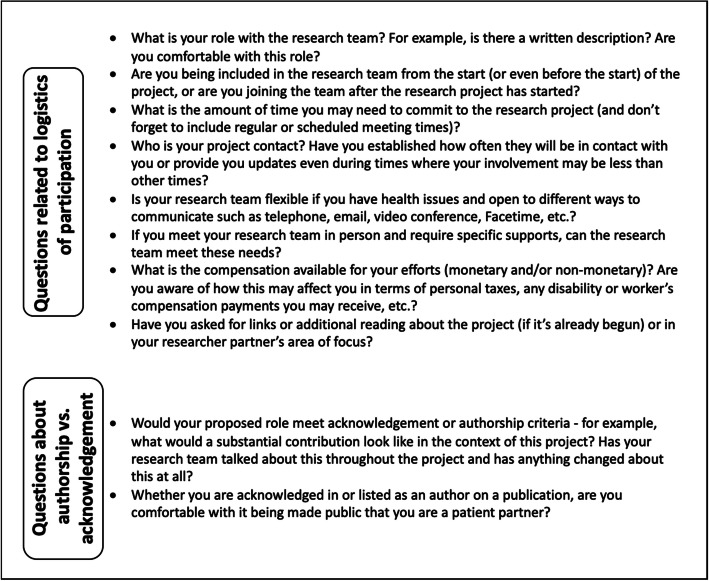
Set of considerations for patient partners to help determine authorship and/or acknowledgement. These are questions that patient partners may wish to ask the research team or their main contact on the research team with respect to helping define expectations as being part of the research team. The aim of considering these questions is to also help determine authorship and/or acknowledgement for their part in the research project

**Fig. 3:**
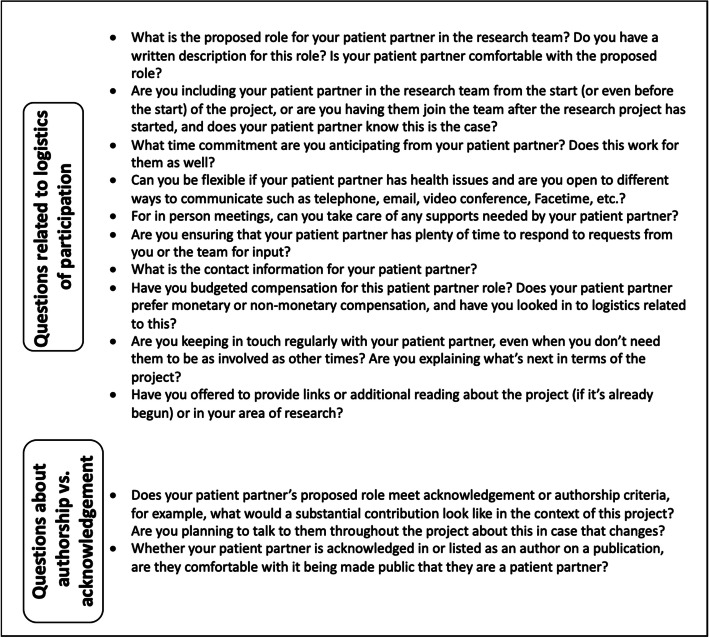
Set of considerations for researchers to help determine authorship and/or acknowledgement for patient partners. These are questions that researchers may wish to consider and discuss with their research project’s patient partner(s) to help define expectations for being part of the research team. The ultimate aim of considering these questions is to also help determine authorship and/or acknowledgement for the patient partner’s part in the research project

Two sets of considerations, one for patient partners (Fig. 2) and one for researchers (Fig. 3), have been created to facilitate decision-making across the range of contribution from acknowledgement (lesser contribution) to authorship (greater contribution). Researchers and patient partners are encouraged to start these discussions early, ideally as soon as they start working together (and this is hopefully at the project outset, but otherwise as soon as the working relationship starts) and throughout the project. While the focus is on authorship and acknowledgement, other relevant considerations are discussed, including research team member roles and compensation [15–17]. Patient partners and researchers are encouraged to review both sets of considerations.

#### Practical advice

Some practical advice exists in the literature about supporting patient partners in an authorship capacity [18]. Additional practical advice offered here is based on the authors’ experiences discussing authorship with researchers and patient partners:

- Provide information to patient partners about the peer review publication process such as timeline, copyright, open access, expectation to revise, and so on (see Fig. 1).
- Involve patient partners as early as possible (from the idea stage ideally) in the research project.
- Co-create a terms of reference document that outlines responsibilities and expectations of all research team members, including patient partners with respect to the research project at the start of the working relationship. This is helpful to set up how the project work will be undertaken together.
- Recognize that it may take time to build relationships between patient partners and research team members. Connections are often easier when face to face (even via a videoconferencing platform) meetings are possible, and when trust builds between team members.
- Discuss the possibilities and responsibilities associated with authorship and acknowledgement early in the project. This helps clarify concepts and expectations for all team members.
- Be frank that authorship is not token and that contributions are required by all authors.
- While patient partners may not be involved in drafting a publication, their intellectual contributions to the project and to the manuscript and data analysis may still warrant authorship.
- Provide patient partners more time and flexibility to contribute to publications. Collect their feedback in writing, by phone, via email or other methods they prefer and that are convenient to them (do not always expect tracked changes back in a typed document).
- Be mindful that not all patient partners have the same resources as your academic research team members, depending on where they live and their own situations. For example, they may have more unreliable internet or phone service, or their email inbox may not have the same capacity as your institutional server. Have patience in understanding the barriers your patient partners may face due to their circumstances.
- Consider that patient partners may have different motivations than others with respect to their participation on the research team. While some patient partners may be more motivated to facilitate change rather than be recognized by publication, other patient partners may be motivated by both the value of facilitating change and being recognized as an author on a publication. In our own experiences, youth patient partners have expressed understanding and seeking the professional value of being recognized as an author and in another case, a caregiver partner expressed that authorship was an important form of recognition to their community.
- Ensure that your patient partners are comfortable with being authors or publicly acknowledged in your publication. Some patient partners may have more sensitivity about being known publicly as having a health condition or health experience than other patient partners. For youth and marginalized individuals this may especially be a sensitive topic to consider.
- For some patient partners, being compensated as a member of the research team may impact their ability to provide additional time and commitment to the research project, in turn impacting whether those contributions would be recognized by authorship of a publication [15–17]. For other patient partners, compensation will not affect their ability and time provided to the research project.

## Conclusions

This guidance has been developed to facilitate conversations about authorship and acknowledgement related to patient engagement and patient-oriented research. While existing recommendations related to authorship and acknowledgement are still relevant, a gap was identified with respect to practical guidance when patient partners are members of research teams. Practical information, context and advice are provided to help the conversations around the topics of authorship and acknowledgement.

## Data Availability

Not applicable.

## Abbreviations

CIHR: Canadian Institutes of Health Research
PE: Patient Engagement
POR: Patient-Oriented Research
